# Gut Microbiome Correlations in Hidradenitis Suppurativa Patients

**DOI:** 10.3390/jcm14145074

**Published:** 2025-07-17

**Authors:** Edyta Lelonek, Piotr K. Krajewski, Jacek C. Szepietowski

**Affiliations:** 1 University Centre of General Dermatology and Oncodermatology, Medical University, 50-556 Wroclaw, Poland; elelonek@gmail.com (E.L.); piotr.krajewski@umw.edu.pl (P.K.K.); 2 Division of Dermatology, Venereology and Clinical Immunology, Faculty of Medicine, Wroclaw University of Science and Technology, 51-377 Wroclaw, Poland; 3 Department of Dermato-Venereology, 4th Military Hospital, 50-981 Wroclaw, Poland

**Keywords:** hidradenitis suppurativa, gut microbiome, microbiota

## Abstract

**Background/Objectives**: Hidradenitis suppurativa (HS) is a chronic autoinflammatory skin disease characterized by recurrent, painful, and persistently draining purulent lesions. Alterations in the composition of the microbiome may be associated with immune dysregulation and HS progression. The objective of this study was to investigate the correlations between the gut microbiome and HS. **Methods**: A total of 80 participants (40 HS patients and 40 healthy controls [HCs]) were included in this study. Each participant filled out a specially designed questionnaire, which included demographics, HS severity, physical characteristics, dietary habits, and gastrointestinal disorders. DNA isolation and sequencing of microbiota were performed from fecal samples collected from each participant. **Results**: No statistically significant difference was observed in the alpha diversity between the microbiota of HS and HC. Nevertheless, HS was found to significantly decrease the chances of, among others, *Collinsella*, *Izemoplasmatales*, *Clostridia*, *Lachnospiraceae*, *eligens* group, *xylanophilum* group, and *Pseudoflavonifractor* occurrence. Conversely, HS significantly increased the chances of *Enterorhabdus*, *Senegalimassilia*, *Gastranaerophilales*, *Candidatus Stoquefichus*, *Erysipelatoclostridiaceae*, *Holdemanella*, *Solobacterium*, *Ruminiclostridium*, [*Eubacterium*] *fissicatena* group, *Angelakisella*, *Comamonas*, and *Enterobacter* occurrence. The logistic regression analysis, performed separately for each genus, showed a significant influence of disease severity (based on the Hurley scale) on the chances of occurrence for the following genera: *Chloroplast* (OR = 5.778), *Dielma* (OR = 5.75), *Eisenbergiella* (OR = 5.75) and *Paludicola* (OR = 5.778). **Conclusions**: In conclusion, our study adds to the growing body of literature on the gut microbiome in HS and provides valuable insights into the specific alterations in microbial occurrence and abundance associated with the disease.

## 1. Introduction

Hidradenitis suppurativa (HS) is a chronic autoinflammatory skin disease characterized by recurrent, painful, and chronically draining purulent lesions primarily affecting intertriginous anatomical sites, such as the axilla, groin, and perineum [1]. Traditionally considered a disease influenced predominantly by local factors, recent research has unveiled a potential role for pathogenic bacteria in the pathogenesis of HS [2]. Alterations in the composition of the microbiome, including the skin and gut microbiota, have been associated with the dysregulation of the immune response and subsequent disease progression in HS [3].

The gut microbiota, comprising trillions of microorganisms residing within the gastrointestinal tract, has garnered increasing attention due to its crucial role in maintaining homeostasis and influencing various aspects of human health and disease [4]. Recent advancements in our understanding of the complex relationship between the skin and gut microbiome have prompted a thorough exploration of the composition and interactions of these microbial communities. Several studies have implicated alterations in the gut microbiota as potential contributors to the pathogenesis of various dermatological conditions, including psoriasis, atopic dermatitis, and acne [5,6,7].

However, the exact mechanisms underlying gut–skin interactions, particularly in the context of HS, have not yet been fully elucidated. While evidence suggests a potential link between HS and dysbiosis of the gut microbiome, the specific microbial alterations and their functional implications in HS remain to be determined [3,8]. Understanding the complex interplay between the gut microbiota and HS could offer novel insights into disease pathogenesis, potential therapeutic targets, and the development of personalized treatment approaches.

The objective of this study was to investigate the correlations between the gut microbiome and HS in order to shed light on the potential associations between gut microbiota and the pathophysiology of this debilitating skin disease. By utilizing advanced sequencing techniques and comprehensive metagenomic analysis, we aimed to characterize the gut microbiota composition in HS patients and compare it to that of healthy individuals. Furthermore, we sought to identify specific microbial taxa and functional pathways that are associated with HS, with a focus on potential pathogenic bacteria and dysbiosis-related mechanisms.

## 2. Materials and Methods

A total of 80 participants were included in this study from October 2021 to March 2023, consisting of 40 patients diagnosed with HS and 40 healthy controls. Patients were consecutively enrolled during routine dermatology consultations, while controls were recruited from volunteers matched for age and sex, without inflammatory skin or gastrointestinal disease. Detailed demographic information, physical characteristics (such as body weight and height), and medical histories, including concomitant diseases and treatments, supplementation, stimulant use, as well as symptoms related to the digestive system and dietary habits, were collected using a specifically designed questionnaire. In female participants, additional information regarding the course of the menstrual cycle was recorded. The selection criteria ensured that the subjects had not received systemic antibiotic therapy (including non-systemic intestinal antibiotics), probiotics, or prebiotics within the last 3 months. Additionally, individuals who were not following a specific diet, such as vegan, vegetarian, or gluten-free, and those without concomitant systemic inflammatory diseases, infections, previous gastrointestinal tract surgery, or malignancy, were eligible for inclusion. The diagnosis of HS was established based on clinical criteria [9]. Disease severity was evaluated using the Hurley staging system and the International Hidradenitis Suppurativa Severity Score System (IHS4) [10,11]. Furthermore, the Polish validated version of the Dermatology Life Quality Index (DLQI) [12,13] questionnaire was administered to assess the impact of HS on patients’ quality of life.

A single fecal sample was collected from each study participant after proper instruction to avoid concomitant drugs, supplements; female participants were instructed to avoid providing samples during menstruation. All enrolled women adhered to this criterion, with 100% compliance confirmed during the screening and scheduling process. Fecal samples were not collected during menstruation to avoid hormonal fluctuations—known to transiently alter gut microbiota composition—and minimize the risk of cross-contamination with vaginal flora during menses. Following collection, the biological material was transferred to OMNIgene tubes • GUT|OM-200 (DNA Genotek, Ottawa, ON, Canada) for preservation and stabilization of the fecal microbiota during transportation and storage. Biological treatment status (e.g., bimekizumab use) was recorded and included as a variable in the logistic regression analyses to evaluate its potential confounding effects on microbiome composition.

DNA was extracted from fecal samples at the Genomed laboratory (Warsaw, Poland) using the Genomic Mini AX Stool kit (A&A Biotechnology), following the manufacturer’s protocol. Next-generation sequencing (NGS) was performed on the isolated DNA. To characterize bacterial and archaeal communities, the hypervariable V3–V4 region of the 16S rRNA gene was amplified using the standard primer pair:

- Forward primer (341F): 5′-CCTACGGGNGGCWGCAG-3′;
- Reverse primer (785R): 5′-GACTACHVGGGTATCTAATCC-3′.

Library preparation and sequencing were conducted according to validated protocols for 16S rRNA amplicon analysis.

PCR amplification was carried out using Q5 Hot Start High-Fidelity 2X Master Mix (New England Biolabs, Inc., Ipswich, MA, USA.) with reaction conditions as recommended by the manufacturer. Sequencing was performed on a MiSeq instrument using paired-end (PE), 2 × 300 nt technology, employing the v3 Illumina kit.

The initial sequencing was performed on the Illumina MiSeq platform using MiSeq Reporter (MSR) software v2.6. Bioinformatic processing, including read quality control, denoising, and taxonomic classification, was conducted using the QIIME 2 software package, with taxonomy assigned against the SILVA 138 reference database. Amplicon sequence variants (ASVs) were inferred using the DADA2 plugin within QIIME 2, which distinguishes true biological sequences from sequencing errors.

Raw reads were filtered and trimmed using the following parameters:

- Forward read truncation length (trunc-len-f): 240 bp;
- Reverse read truncation length (trunc-len-r): 200 bp;
- Trimming at start (trim-left-f/r): 0 bp;
- Maximum expected errors (maxEE): 2 for forward, 5 for reverse reads;
- Truncation quality score (truncQ): 2;
- Minimum overlap for merging: 12 bp;
- Chimera detection method: consensus (removeBimeraDenovo).

These parameters were selected based on the quality profile of the reads to maximize retention of high-quality sequences while minimizing error propagation. Chimeric sequences were removed during denoising using the consensus method [14,15,16,17,18,19,20,21,22].

The statistical analysis was performed using R software, version 4.1.3, which provides a comprehensive environment for data analysis and visualization [23]. Statistical analysis was performed to evaluate the data obtained from the questionnaires, clinical assessments, and sequencing results. To compare the values of qualitative variables between groups, the chi-square test (with Yates’ correction for 2 × 2 tables) or Fisher’s exact test was performed, and, for the evaluation of the values of quantitative variables between two groups, the Mann–Whitney U test was used. Linear regression analysis was used to assess the multiple factors influencing the quantitative variable. Results are presented as parameter estimates of the regression model, along with their corresponding 95% confidence intervals.

Logistic regression analysis was employed to analyze the multiple factors affecting a binary variable. The results were presented as odds ratios (OR) with their corresponding 95% confidence intervals. A significance level of 0.05 was used for all statistical tests. Therefore, *p*-values below 0.05 were considered indicative of statistically significant associations. For genus-level taxa, we re-estimated logistic regression models predicting genus presence (binary outcome), adjusting for BMI and smoking alongside disease status. Model stability for rare taxa was ensured by using penalized (Firth) correction when necessary, and we planned LASSO penalization if overfitting was evident. Alpha diversity (Shannon index and observed richness) was compared between groups using nonparametric tests; these diversity analyses were repeated with BMI and smoking as covariates to confirm that the lack of difference was not due to confounding.

This study was conducted in accordance with the ethical principles outlined in the Declaration of Helsinki. Ethical approval was obtained from the Ethics Committee at the Medical University in Wroclaw, Poland (No. 100/2023). All participants provided informed written consent prior to their participation in this study. All patient data were anonymized using unique alphanumeric codes, and all personal identifiers were removed prior to analysis. Data were stored on secure, password-protected servers accessible only to the research team.

## 3. Results

The main participants’ characteristics are presented in Table 1.

**Figure 1:**
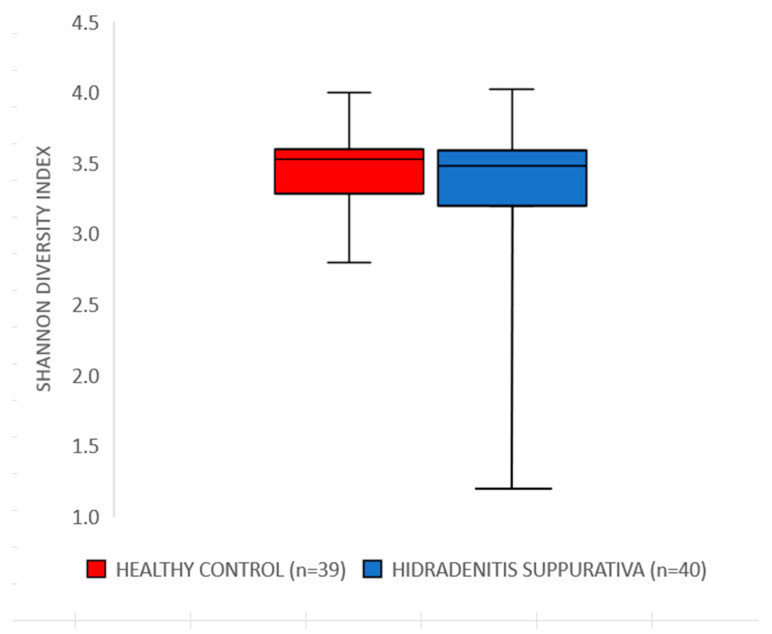
Differences in the Shannon diversity index between hidradenitis suppurativa patients and healthy controls.

The observed alpha-diversity and Shannon diversity index of the gut microbiome between individuals diagnosed with HS and healthy volunteers showed no statistically significant differences between the groups for both the number of species present in the sample (*p* = 0.41) and the Shannon diversity index (*p* = 0.346) (Figure 1). Supplementary Figure S1 illustrates a distinct shift in gut microbiota composition between HS patients and healthy controls.

**Figure 2:**
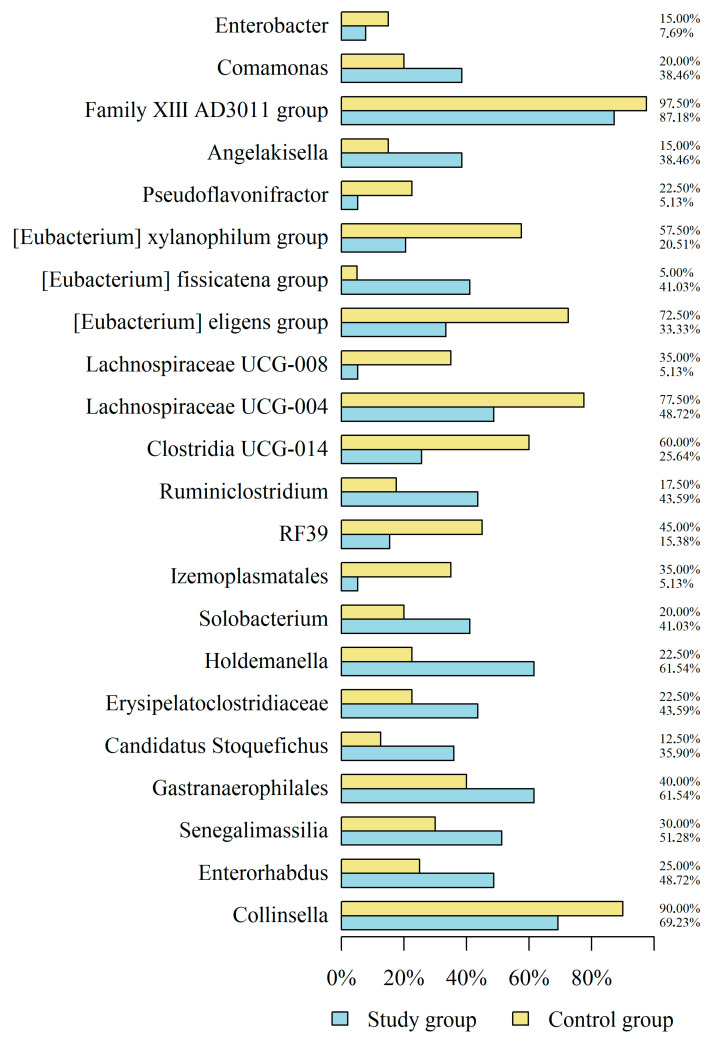
The 22 most common bacterial genera identified in the study and control groups.

Furthermore, the investigation comparing the gut microbiome composition in both groups yielded non-significant distinctions, as determined by PERMANOVA statistical analyses (*p* > 0.05). Figure 2 displays the detection frequency of the 22 most common bacterial genera in HS patients and controls, defined by the highest mean relative abundance across all samples, offering a comparative overview of their presence. Due to the sparsity of many taxa across samples, Figure 2 is based on detection frequency (presence across individuals) rather than relative abundance, which would have excluded low-prevalence genera or necessitated imputation.

Additional inclusion of BMI and smoking as covariates did not change observed species richness and Shannon index (adjusted *p* > 0.05). Likewise, β-diversity analysis revealed no significant separation of HS and control microbiota after accounting for BMI and smoking (PERMANOVA *p* > 0.1).

**Figure 3:**
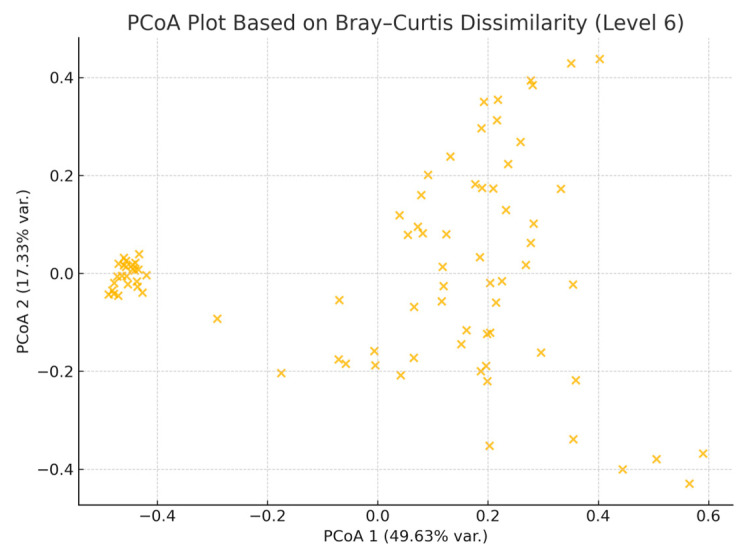
Principal Coordinates Analysis (PCoA) of gut microbiota based on Bray–Curtis dissimilarity. Each point represents a sample.

To visualize differences in microbial community composition between groups, we performed Principal Coordinates Analysis (PCoA) based on Bray–Curtis dissimilarity. The resulting plot suggested some separation between HS patients and healthy controls; however, this was not statistically significant based on PERMANOVA. The first two principal coordinates explained 49.63% and 17.33% of the total variance, respectively (Figure 3).

The analysis scrutinized the impact of HS on the occurrence of various genera within the gut microbiome while controlling for BMI and smoking status. The results revealed significant associations between HS and specific genera. HS was associated with significantly lower odds of detecting certain taxa, including *Collinsella*, *Izemoplasmatales*, *Clostridia UCG-014*, *Lachnospiraceae UCG-004*, *Lachnospiraceae UCG-008*, [*Eubacterium*] *eligens* group, [*Eubacterium*] *xylanophilum* group, and *Pseudoflavonifractor* occurrence. Conversely, HS significantly increased the chances of *Enterorhabdus*, *Senegalimassilia*, *Gastranaerophilales*, *Candidatus Stoquefichus*, *Erysipelatoclostridiaceae*, *Holdemanella*, *Solobacterium*, *Ruminiclostridium*, [*Eubacterium*] *fissicatena* group, *Angelakisella*, *Comamonas*, and *Enterobacter* occurrence (Table 2).

Additionally, the abundance of *Desulfovibrionales*, *Clostridia*, and *Opitutales* was significantly influenced by the HS (regression parameter 102.907, 6.021, and −20.408, respectively). The logistic regression analysis, performed separately for each genera, showed a significant influence of disease severity (based on the Hurley scale) on the chances of occurrence for the following genera: *Chloroplast* (OR = 5.778), *Dielma* (OR = 5.75), *Eisenbergiella* (OR = 5.75), and *Paludicola* (OR = 5.778) (Table 3).

Furthermore, we found that, within the HS group, BMI had a substantial impact on the microbiome. Higher BMI was associated with reduced odds of occurrence for a broad array of genera. For instance, *Enterorhabdus*, *Senegalimassilia*, *Coprobacter*, *Gastranaerophilales*, *Desulfovibrio*, *Candidatus Stoquefichus*, *Erysipelatoclostridiaceae*, *Erysipelatoclostridium*, *Dielma*, *Holdemanella*, *Christensenellaceae* R-7 group, *Ruminiclostridium*, *UCG-002*, *Anaerotruncus*, *Candidatus Soleaferrea*, *DTU089*, [*Eubacterium*] *siraeum* group, *UCG-010*, [*Clostridium*] *methylpentosum* group, *Phascolarctobacterium*, *Comamonas*, and Family XIII *UCG-001* all demonstrated a negative association with BMI (ranging from 10.3% to 29.7%). Contrarily, certain genera showed a positive association with BMI. *Merdibacter*, *Lactobacillus*, *Gemella*, *Dialister*, and *Veillonella* exhibited an increased likelihood of occurrence with higher BMI (ranging from 12.2% to 26.1%) (Table 4).

Logistic regression evaluations conducted individually for each genus in the control group revealed that BMI significantly affects the probability of occurrence for specific genera. An increase in BMI was associated with a higher likelihood of *Prevotella* (OR = 1.197) and *Lachnospiraceae* UCG-008 (OR = 1.213), with each kg/m^2^ increase in BMI raising the chances of these genera by 19.7% and 21.3%, respectively. In contrast, a higher BMI was associated with a decreased likelihood of *Intestinimonas* (OR = 0.854), with each kg/m^2^ increase in BMI reducing the likelihood of its occurrence by 14.6%.

To control for the influence of biological therapy, we conducted subgroup analyses comparing patients receiving bimekizumab with those not on biologics, and incorporated treatment status into logistic regression models assessing microbial taxa occurrence. The biological treatment outcomes on the likelihood of facilitating the occurrence of particular bacterial genera within the microbial community demonstrated a substantial impact. The evaluation exposed that biological usage significantly decreased the likelihood of *Collinsella*, *Enterorhabdus*, *Senegalimassilia*, *Slackia*, *Gastranaerophilales*, *Desulfovibrio*, *Erysipelotrichaceae UCG-003*, *Holdemanella*, *Defluviitaleaceae UCG-011*, *Marvinbryantia*, *Tyzzerella*, [*Eubacterium*] *eligens* group, [*Ruminococcus*] *gauvreauii* group, [*Eubacterium*] *siraeum* group, [*Clostridium*] *methylpentosum* group, *Peptococcus*, *Oxalobacter*, and *Victivallis*, ranging from 77.0% to 97.6%. Conversely, biologic therapy was associated with increased odds of detecting *Adlercreutzia*, *Paraprevotella*, *Prevotella*, *Merdibacter*, *RF39*, *Clostridia UCG*-014, *Lachnospiraceae UCG*-*004*, *CAG*-352, [*Eubacterium*] brachy group, *Intestinibacter*, and *Dialister*, ranging from 4.343 to 11.5 times, with biological treatment.

The logistic regression analysis across different genera indicated a significant impact of the DLQI score on the likelihood of occurrence for certain microbes. Specifically, the following:

- An increase in the DLQI score was associated with a decreased probability of encountering *Agathobacter* and the [*Eubacterium*] *eligens* group, with odds ratios of 0.878 and 0.789, respectively;
- Conversely, a higher DLQI score correlated with an increased probability of *Comamonas* presence, with an odds ratio of 1.166.

## 4. Discussion

The notion of a “gut–skin axis” in HS is reinforced by growing evidence that gut dysbiosis associations suggest a potential role and immune dysregulation in this disease. Multiple studies, though heterogeneous, suggest HS is accompanied by an imbalance favoring pro-inflammatory microbes over beneficial commensals. For instance, *Proteobacteria* (e.g., *Enterobacteriaceae*) and certain *Actinobacteria* tend to be enriched in HS gut communities, whereas anti-inflammatory *Firmicutes* and *Bacteroidetes* populations may be reduced. Such shifts are characteristic of a dysbiotic state that can impair the gut’s immunoregulatory functions [24]. Notably, a reduced overall microbial diversity has been observed in several HS cohorts [25,26], aligning with patterns seen in other inflammatory disorders where lower diversity correlates with disease activity. Even within studies, specific taxa show inconsistent patterns. *Bilophila*—a sulfide-producing pathobiont—was found enriched in several HS cohorts [25,27], as was *Ruminococcus gnavus*, a species linked to inflammatory bowel disease [26]. Yet, in a pediatric HS population, *Faecalibacterium prausnitzii*, typically depleted in inflammatory disorders, was unexpectedly elevated [27]. Likewise, *Veillonella* was decreased in one study [25], but was found to be increased in a subset of HS patients exhibiting a Crohn-like microbiota profile [26]. Beta-diversity results have also been inconsistent; while some studies report clear compositional differences between HS and control groups [28], others found no distinct clustering [27]. These inconsistencies likely reflect differences in sequencing methodology, study size, dietary and lifestyle factors, and participant demographics such as age or comorbidities.

This emerging picture supports the concept that HS involves not merely localized follicular occlusion but also systemic alterations in host–microbe homeostasis in the gut. Differences between studies remain, yet a common theme is an imbalance in microbial composition, which may set the stage for aberrant immune signaling in HS [24]. Future studies may help determine whether these microbial patterns could contribute to biomarker development or therapeutic targeting in HS. Future research using standardized protocols and larger cohorts is needed to resolve these discrepancies and clarify whether dysbiosis is a contributing cause or a downstream effect of systemic inflammation in HS.

A key mechanism by which a dysbiotic gut microbiome might exacerbate HS is through the release of microbial products that fuel systemic inflammation. Overgrowth of Gram-negative bacteria (e.g., *Enterobacter* or *Escherichia coli*) can increase luminal lipopolysaccharide (LPS), peptidoglycan, and other pathogen-associated molecules, which translocate across a perturbed intestinal barrier and trigger toll-like receptors and other pattern recognition receptors [24,29]. This cascade promotes pro-inflammatory cytokines (TNF-α, IL-1β, IL-6) that are known to be elevated in HS lesions [24]. At the same time, the loss of beneficial microbes may deprive the host of important anti-inflammatory signals. Many taxa depleted in our HS patients (such as *Lachnospiraceae* family members and *Eubacterium* spp.) are short-chain fatty acid (SCFA) producers that generate butyrate and other metabolites crucial for intestinal regulatory immune responses. Diminished SCFA levels can lead to reduced induction of anti-inflammatory cytokines like IL-10 and TGF-β and a breakdown of mucosal tolerance [30]. In HS, this could tilt the immune system further toward a pro-inflammatory Th1/Th17-skewed state. Moreover, gut dysbiosis may compromise gut barrier integrity (“leaky gut”), allowing endotoxins and microbial antigens to enter circulation and perpetuate systemic inflammation [29]. Although direct evidence of increased gut permeability in HS is still limited, these putative mechanisms mirror those in other chronic inflammatory conditions and provide a plausible link between gut microbiota alterations and HS’s exaggerated immune activation.

Our findings of taxa-specific differences in HS gain further context when compared with other research. The decreased abundance of genera such as *Collinsella*, *Lachnospiraceae UCG*-004/008, [*Eubacterium*] *eligens* and *xylanophilum* groups, and *Pseudoflavonifractor* in HS patients is notable. Many of these belong to families (e.g., *Coriobacteriaceae*, *Lachnospiraceae*, *Ruminococcaceae*) that are instrumental in fermenting dietary fibers and producing SCFAs that reinforce gut barrier function and immune homeostasis [27]. Their underrepresentation in HS could thus have functional significance, implying a loss of immunoregulatory capacity in the gut ecosystem. Interestingly, one recent Mendelian randomization study identified low abundance of certain commensals as potentially protective against HS, suggesting a causal relationship: a higher relative abundance of the butyrate-producing family *Porphyromonadaceae* and the *clostridial Family XI* (*Clostridium cluster XI*) was associated with reduced HS risk (odds ratios 0.29 and 0.67, respectively) [31]. These taxa include bacteria that may curb inflammation, reinforcing the idea that their depletion in HS could remove a critical check on immune activation. In contrast, our data and other studies did not find a consistent decrease in *Faecalibacterium prausnitzii*—a prominent anti-inflammatory commensal often diminished in Crohn’s disease and psoriasis—among HS patients [32]. Eppinga et al. [32] noted that, while *F. prausnitzii* was markedly depleted in psoriasis (with or without IBD) and in IBD alone, its levels in HS without IBD remained comparable to controls. This suggests that the gut microbiome signature of HS, albeit dysbiotic, might be distinct from those of other inflammatory skin disorders, potentially indicating different disease-specific microbial drivers or compensatory mechanisms.

Conversely, HS patients in our cohort showed increased occurrence of several genera with known or suspected pro-inflammatory roles, paralleling findings from other investigations. We observed higher representation of *Proteobacteria* such as *Enterobacter* and *Comamonas*, which are opportunistic organisms whose endotoxins (e.g., LPS) can promote systemic inflammation [33]. In line with this, McCarthy et al. [32] reported an enrichment of *Escherichia*/*Shigella* and *Enterococcus* in the gut of a subset of HS patients. Similarly, Actinobacteria like *Eggerthella* (which our results implicate via the genus *Enterorhabdus*, a related member of *Eggerthellaceae*) have been found at higher abundance in HS and are known to proliferate in inflammatory gut conditions. Of particular interest, Firmicutes members associated with inflammation were also elevated. The *Erysipelotrichaceae* family (notably *Clostridium ramosum*, recently reclassified as *Erysipelatoclostridium ramosum*) and certain *Ruminococcaceae* (e.g., *Ruminococcus gnavus*) have each been identified as key discriminative taxa in HS, frequently co-occurring with Crohn’s-like dysbiosis. These organisms are capable of degrading mucins and releasing pro-inflammatory metabolites and have been linked to increased TNF-α production in IBD models. In fact, multiple studies have highlighted that the HS gut microbiome can mirror features of IBD: *R. gnavus* and *C. ramosum* are consistently enriched in HS and Crohn’s disease alike, contributing to a Th1-polarized cytokine milieu (elevated TNF-α, IL-12) in both conditions [34].

A recent study by Cronin et al. [35] found that approximately 40% of HS patients exhibit a “Crohn-like” gut microbiota configuration, characterized by an overabundance of pathogenic genera, such as *Enterococcus*, *Veillonella*, and *Escherichia*/*Shigella*, and a pronounced depletion of beneficial *Faecalibacterium*. Intriguingly, those HS patients with this dysbiotic cluster had significantly lower levels of the anti-inflammatory mediator GAS6 and showed an inverse correlation between IL-12 (a pro-inflammatory cytokine) and the abundance of health-associated gut genera. Such findings imply that a subset of HS patients may carry a high-risk microbiome profile that amplifies systemic inflammation and potentially predisposes them to comorbid conditions like IBD. In contrast, the remaining ~60% of HS patients in that study harbored more normal gut microbiota resembling healthy controls, illustrating the heterogeneity within the HS population. This heterogeneity could explain why not all studies detect the same microbial shifts—some patients may have pronounced dysbiosis, while others do not, possibly reflecting differences in disease severity, treatment history, or genetic backgrounds.

It is also illuminating to compare HS to other inflammatory or dermatologic diseases with respect to gut microbiome changes. For example, in rheumatoid arthritis (RA)—another immune-mediated condition—patients show an expansion of certain gut Actinobacteria like *Collinsella*, which correlates with increased intestinal permeability and elevated IL-17-driven inflammation. Experimentally, high *Collinsella* can worsen arthritis severity by degrading epithelial tight junctions and promoting Th17 responses [36]. In our HS cohort, however, *Collinsella* was less prevalent than in controls, suggesting that the microbial drivers of inflammation may differ between HS and RA. Likewise, gut dysbiosis in psoriasis has been documented (e.g., reduced *F. prausnitzii* and other butyrate-producers), yet HS appears to diverge in specific taxa while sharing the broader theme of a pro-inflammatory microbiome [32]. These comparisons highlight that, while gut microbiome perturbations are a recurring feature across chronic inflammatory disorders, the composition of those perturbations is disease-specific. HS’s microbial signature might thus interact with its unique pathogenic pathways in ways distinct from purely autoimmune diseases.

The clinical implications of gut microbiome alterations in HS are far-reaching. Our observation that certain intestinal genera correlated with Hurley stage suggests that the degree of dysbiosis may track with HS progression or burden. It is tempting to speculate that, in more severe HS, a more pronounced loss of beneficial bacteria and gain of pro-inflammatory species creates a systemic environment conducive to frequent flares, poor wound healing, and heightened pain. Indeed, HS patients with metabolic comorbidities (obesity, insulin resistance) might experience a compounded effect, as obesity itself alters gut microbiota and promotes a chronic inflammatory state [24]. Higher body mass index has been linked to shifts in gut flora (e.g., favoring inflammation-associated taxa), and our data confirmed that BMI influences the presence of specific microbes in HS. This intersection between metabolic factors and microbiome is important; it raises the possibility that part of the HS–obesity link may be mediated through microbiome-driven inflammation. It is also possible that some of the microbial differences observed in HS patients may be attributable to obesity as a comorbid condition, rather than HS itself, given the known influence of BMI on gut microbiota composition. This overlap highlights the need for future studies using BMI-matched controls or stratified analyses to better isolate HS-specific microbial signatures.

Similarly, the noted association between DLQI and certain bacterial genera hints that dysbiosis might contribute to the symptomatic burden of HS. Patients with worse quality-of-life scores could have more aberrant microbiomes, though causality is unclear—severe disease could both result from and further perpetuate microbiome disturbances via stress, diet changes, or antibiotic exposure.

Importantly, treatments for HS may themselves impact the gut microbiome, and vice versa. Chronic antibiotic therapy—a common HS management strategy—can profoundly perturb gut microbial communities, reducing diversity and selectively depleting susceptible taxa [35]. This confounding factor must be considered when interpreting microbiome studies, as past antibiotic use could mask or mimic disease-related changes. On the other hand, effective systemic treatments might help restore microbiome balance indirectly. For example, recent evidence indicates that TNF-α inhibitor therapy (adalimumab) in HS is associated with increased fecal SCFA levels, reflecting a rebound of SCFA-producing bacteria in treated patients [37]. Bimekizumab’s inhibition of IL-17A and IL-17F could have downstream effects on the gut microbiome that are relevant to HS. In related diseases, IL-17 blockade has been shown to alter intestinal microbial composition, for example, by reducing SCFA-producing Firmicutes (such as *Blautia* and *Roseburia*) and increasing Bacteroidetes (e.g., *Bacteroides stercoris*, *Parabacteroides merdae*) [38], alongside an overgrowth of Candida in some patients [39]. These dysbiotic shifts reflect the loss of IL-17-mediated mucosal defense and have been linked to subclinical gut inflammation [39,40]. While direct evidence in HS is absent, this suggests that bimekizumab may modulate the gut microbiota, potentially influencing systemic inflammatory tone and treatment response. This finding suggests that quelling inflammation in HS (through biologics or other means) may create a more permissive environment for beneficial gut microbes to thrive, breaking the vicious cycle of dysbiosis and inflammation.

Conversely, it also raises the intriguing possibility that microbiome-targeted interventions could serve as adjunct therapies in HS. Indeed, probiotics and prebiotics have been proposed as a means to modulate the gut–skin axis in HS. By introducing beneficial strains or fostering their growth, probiotic supplementation might help rebalance the intestinal microbiota and thereby reduce systemic inflammation. Early clinical anecdotes and parallel experiences in diseases like atopic dermatitis and acne support this concept, though robust trials in HS are lacking [41,42,43]. Given the chronic inflammatory nature of HS, such microbiome-directed strategies are compelling. While much research is still needed, these perspectives open new avenues where diet, microbiota, and immunity converge in HS management.

Finally, our results regarding *Desulfovibrionales*, *Clostridia*, and *Opitutales* further emphasize the link between HS and gut microbial dysregulation. *Desulfovibrio* species (order *Desulfovibrionales*) are sulfate-reducing bacteria that generate hydrogen sulfide, a compound known to disrupt the intestinal barrier and incite mucosal inflammation. Their enrichment in HS patients could thus contribute to an inflammatory gut milieu. Likewise, shifts in certain *Clostridia* subclasses and in *Opitutales* (an order of *Verrucomicrobia*) have been observed in inflammatory conditions; for example, murine models of colitis show expansion of specific *Clostridial* groups and *Verrucomicrobia* concomitant with disease [29]. The significant alterations we observed in these higher taxonomic groups in HS underscore that microbiome changes are not confined to a few genera but span multiple bacterial lineages associated with inflammation. Taken together, these insights paint a more nuanced picture of how the gut microbiome may influence HS pathogenesis, severity, and treatment response. They highlight that HS, traditionally viewed as a skin-localized disorder, likely involves a systemic component wherein gut microbes and their metabolites modulate immune pathways relevant to disease expression. Continued research—especially longitudinal and interventional studies—is warranted to unravel the precise mechanisms at play and to determine whether manipulating the gut microbiota can favorably alter the course of HS. In the meantime, recognizing the gut–skin connection in HS adds a further dimension to our understanding of this complex disease and points toward holistic therapeutic strategies that address both cutaneous lesions and the underlying systemic imbalances.

It is important to acknowledge some limitations of our study. It was a single-center study with a relatively small sample size, which may affect the generalizability of the results. Further research on a more diverse population should be conducted in order to fully understand the microbiome alterations.

Additionally, the cross-sectional design limits our ability to establish causal relationships between HS and gut microbiome alterations. Longitudinal studies with larger cohorts are needed to validate our findings and provide a more comprehensive understanding of the complex interactions between HS and the gut microbiota.

While our analyses adjusted for BMI and smoking, it remains challenging to fully disentangle microbiome alterations that are specific to HS from those that may be driven by obesity or metabolic comorbidities. Given the well-documented impact of obesity on gut microbial composition and systemic inflammation, future studies should consider stratifying participants by BMI categories. Such stratification would help to clarify whether the observed dysbiosis patterns are intrinsic to HS pathophysiology or reflect overlapping obesity-related microbiome shifts. A more granular approach to cohort design—such as including lean HS patients and obese controls—may provide clearer insights into disease-specific microbial signatures.

In addition to BMI and smoking, other potential confounders such as psychological stress, diet, and medication use are known to influence both microbiome composition and immune function and may contribute to inter-individual variability. Future studies should incorporate standardized tools to quantify psychosocial stress (e.g., Perceived Stress Scale) and detailed dietary assessments (e.g., food frequency questionnaires or dietary logs). Collecting these data would allow for more robust multivariate analyses and improve the ability to isolate HS-specific microbiome alterations from those related to lifestyle, nutrition, or emotional stress.

Despite efforts to minimize confounding through strict inclusion criteria and statistical adjustments, we acknowledge that other unmeasured variables—such as stress levels, sleep patterns, unreported dietary differences, and physical activity—could have influenced the gut microbiota composition. These factors, although difficult to fully control in observational studies, may represent meaningful sources of variability.

We acknowledge that our study did not uncover a specific molecular mechanism linking the gut microbiome to skin inflammation in HS. However, we respectfully emphasize that continued exploration of the gut–skin axis in HS remains scientifically warranted and clinically significant, even if the precise mechanisms are not yet fully elucidated. Emerging evidence suggests the gut–skin microbiota as an important contributor to inflammatory skin diseases (including HS) and as a potential direction for therapeutic investigation, despite our still-evolving understanding of the underlying pathways. In other words, the absence of a definitive mechanism does not diminish the value of uncovering microbial associations that may inform future research.

## 5. Conclusions

In conclusion, our study adds to the growing body of literature on the gut microbiome in HS and provides valuable insights into the specific alterations in microbial occurrence and abundance associated with the disease. Although no significant differences were observed in overall microbial diversity and composition, our findings highlight the significant associations between HS and specific bacterial genera, as well as the influence on the abundance of certain bacterial groups. These deductions provide valuable insights into the intricate relationship between HS, disease severity, BMI, biological treatment, quality of life, and the composition of specific bacterial taxa. Further research is warranted to elucidate the underlying mechanisms and functional implications of these findings, which may ultimately contribute to the development of novel diagnostic and therapeutic approaches for HS.

## Figures and Tables

**Table 1:** Group characteristics.

Parameter		Group		*p*
		Study Group (N ^1^ = 40)	Control Group (N = 40)	
Age [years]	mean ± SD ^2^	39.02 ± 11.69	43.23 ± 18.18	*p* = 0.494
	median	40	39.5	
	quartiles	32–46	27–60.25	
BMI ^3^ [kg/m^2^]	mean ± SD	30.36 ± 6.77	25.05 ± 4.77	*p* < 0.001 *
	median	28.73	24.75	
	quartiles	25.66–32	20.59–28.83	
WHR ^4^	mean ± SD	0.87 ± 0.11	0.81 ± 0.09	*p* = 0.004 *
	median	0.86	0.81	
	quartiles	0.79–0.89	0.74–0.85	
Gender	Female	18 (45.00%)	27 (67.50%)	*p* = 0.071
	Male	22 (55.00%)	13 (32.50%)	
Residence	Rural	11 (27.50%)	8 (20.00%)	*p* = 0.599
	Urban	29 (72.50%)	32 (80.00%)	
Education	Primary	2 (5.00%)	1 (2.50%)	*p* = 0.005 *
	Vocational	8 (20.00%)	2 (5.00%)	
	Secondary	18 (45.00%)	10 (25.00%)	
	Higher	12 (30.00%)	27 (67.50%)	
Occupational status	Student	5 (12.50%)	2 (5.00%)	*p* = 0.003 *
	Employed	28 (70.00%)	30 (75.00%)	
	Unemployed	6 (15.00%)	0 (0.00%)	
	Retired	1 (2.50%)	8 (20.00%)	
Tobacco smoking	No	17 (42.50%)	33 (82.50%)	*p* = 0.001 *
	Yes	23 (57.50%)	7 (17.50%)	
Alcohol consumption	No	12 (30.00%)	10 (25.00%)	*p* = 0.773
	Yes	5 (12.50%)	7 (17.50%)	
	Occasionally	23 (57.50%)	23 (57.50%)	
Drug use	Never	29 (72.50%)	36 (90.00%)	*p* = 0.165
	Occasionally	4 (10.00%)	1 (2.50%)	
	In the past	7 (17.50%)	3 (7.50%)	
Diet	No	19 (47.50%)	23 (57.50%)	*p* = 0.502
	Yes	21 (52.50%)	17 (42.50%)	
Physical activity	Sedentary	8 (20.00%)	3 (7.50%)	*p* = 0.041 *
	Low	13 (32.50%)	11 (27.50%)	
	Moderate	13 (32.50%)	12 (30.00%)	
	Active	6 (15.00%)	7 (17.50%)	
	Very active	0 (0.00%)	7 (17.50%)	
Duration of the disease [months]	mean ± SD median quartiles	93.92 ± 70.94 72 54.25–99	- - -	
Biologic treatment	Adalimumab Bimekizumab other	013 (32.5%) 0	0 0 0	
IHS4 ^5^ [points]	mean ± SD median quartiles	21.52 ± 16.3 18 11–25.25	- - -	
DLQI ^6^ [points]	mean ± SD median quartiles	6.7 ± 5.82 5.5 2–8.25	- - -	
Hurley Staging	Stage II Stage III	30 (75%) 10 (25%)	- -	

^1^ N—number of participants; ^2^ SD—standard deviation; ^3^ BMI—body mass index; ^4^ WHR—waist hip ratio; ^5^ IHS4—international hidradenitis suppurativa severity score; ^6^ DLQI—dermatology life quality index; *—statistically significant.

**Table 2:** Differences in microbiome order-level abundance between study and control groups.

Order	Parameter	95% CI ^1^		*p*
*Desulfovibrionales*	102,907	1681	204,133	0.05 *
*Clostridia*	6021	1753	10,289	0.007 *
*Opitutales*	−20,408	−37,498	−3319	0.022 *

^1^ CI—confidence interval. *—statistically significant.

**Table 3:** Logistic regression analysis (separate regression for each genus) depending on the stage of disease (Hurley staging).

Genus	OR ^1^	95% CI ^2^		*p*
*Chloroplast*	5.778	1.014	32.928	0.048
*Dielma*	5.75	1.218	27.138	0.027
*Eisenbergiella*	5.75	1.218	27.138	0.027
*Paludicola*	5.778	1.014	32.928	0.048

^1^ OR—odds ratio; ^2^ CI—confidence interval.

**Table 4:** The chances of developing the following genera depending on the BMI of HS patients assessed with the logistic regression analysis.

Genus	OR ^1^	95% CI ^2^		*p*
*Enterorhabdus*	0.703	0.547	0.904	0.006
*Senegalimassilia*	4.181	1.318	13.261	0.015
*Dielma*	0.868	0.763	0.987	0.03
*Coprobacter*	0.833	0.728	0.953	0.007
*Gastranaerophilales*	0.863	0.763	0.976	0.019
*Desulfovibrio*	0.83	0.714	0.965	0.015
*Candidatus Stoquefichus*	0.847	0.72	0.995	0.043
*Erysipelatoclostridiaceae*	0.775	0.635	0.945	0.012
*Erysipelatoclostridium*	0.891	0.795	1	0.049
*Holdemanella*	0.831	0.722	0.957	0.01
*Christensenellaceae R-7 group*	0.874	0.769	0.993	0.039
*Ruminiclostridium*	0.775	0.635	0.945	0.012
*UCG-002*	0.872	0.771	0.986	0.029
*Anaerotruncus*	0.887	0.789	0.997	0.044
*Candidatus Soleaferrea*	0.873	0.766	0.995	0.042
*DTU089*	0.867	0.768	0.979	0.021
[*Eubacterium*] *siraeum group*	0.814	0.701	0.945	0.007
*UCG-010*	0.863	0.766	0.973	0.016
[*Clostridium*] *methylpentosum group*	0.849	0.741	0.973	0.019
*Phascolarctobacterium*	0.883	0.79	0.988	0.03
*Family XIII UCG-001*	0.896	0.803	1	0.049
*Comamonas*	0.737	0.582	0.934	0.011
*Merdibacter*	1.122	1.006	1.251	0.039
*Lactobacillus*	1.224	1.052	1.425	0.009
*Gemella*	1.261	1.001	1.587	0.049
*Dialister*	1.126	1.008	1.257	0.035

^1^ OR—odds ratio; ^2^ CI—confidence interval.

## Data Availability

The data supporting this study are available in the following online repository: https://doi.org/10.6084/m9.figshare.26083525.v1.

## Supplementary Materials

The following supporting information can be downloaded at: https://www.mdpi.com/article/10.3390/jcm14145074/s1, Figure S1: Comparative gut microbiome composition in HS patients vs. controls.

## Abbreviations

The following abbreviations are used in this manuscript:

HS	Hidradenitis suppurativa
HC	Healthy controls
OR	Odds Ratio
PCR	Polymerase Chain Reaction
DLQI	Dermatology Life Quality Index
DNA	Deoxyribonucleic acid
BMI	Body Mass Index
TNF	Tumor Necrosis Factor
IBD	Inflammatory Bowel Disease
RA	Rheumatoid Arthritis
