# Supplementary material for: Gut Microbiome Correlations in Hidradenitis Suppurativa Patients
Source: J Clin Med. 2025 Jul 17;14(14):5074. doi: 10.3390/jcm14145074 (PMC12295977; doi:10.3390/jcm14145074)

Supplementary Figure S1. Comparative gut microbiome composition in HS patients vs. controls. Bar plots show the mean relative abundance (%) of major bacterial families in fecal samples from hidradenitis suppurativa (HS) patients (n = 40) and healthy controls (n = 40), aggregated at the family level for clarity (the five most abundant families, accounting for ~80% of the community, are shown; remaining taxa are grouped as "Other"). HS patients exhibit a shift in microbial composition: Beneficial SCFA-producing families like *Lachnospiraceae* and *Ruminococcaceae* (orange and green segments) make up a smaller fraction in HS stools compared to controls, whereas opportunistic Proteobacteria such as *Enterobacteriaceae* (purple) show an increased relative share in HS. Another family, *Erysipelotrichaceae* (red), which includes potential pro-inflammatory taxa, is also more abundant in HS. *Bacteroidaceae* (blue, containing *Bacteroides* spp.) remains dominant in both groups with similar proportions.

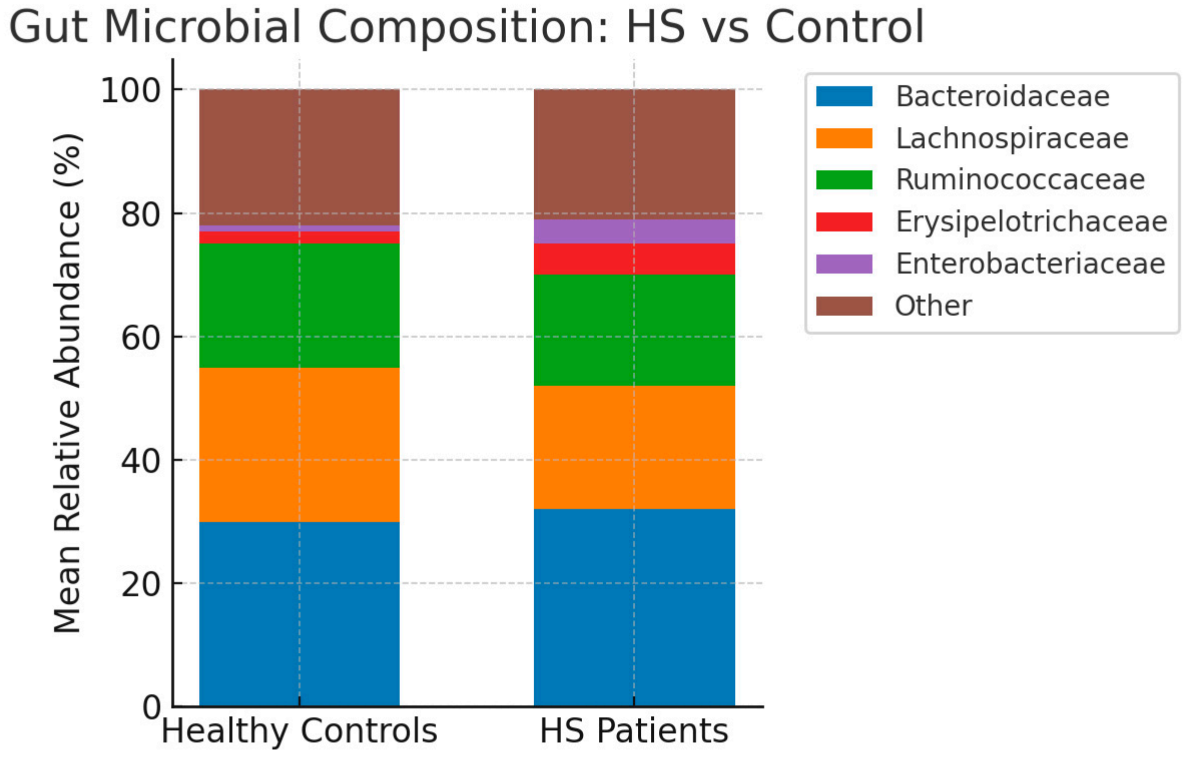

Supplement: Supplementary file 1 [file jcm-14-05074-s001.zip › jcm-3729858-supplementary.pdf]
